# Complex Spatial Dynamics of Oncolytic Viruses In Vitro: Mathematical and Experimental Approaches

**DOI:** 10.1371/journal.pcbi.1002547

**Published:** 2012-06-14

**Authors:** Dominik Wodarz, Andrew Hofacre, John W. Lau, Zhiying Sun, Hung Fan, Natalia L. Komarova

**Affiliations:** 1 Department of Ecology and Evolutionary Biology, University of California, Irvine, California, United States of America; 2 Department of Mathematics, Rowland Hall, University of California, Irvine, California, United States of America; 3 Department of Molecular Biology and Biochemistry, Cancer Research Institute, University of California Irvine, California, United States of America; University of Texas at Austin, United States of America

## Abstract

Oncolytic viruses replicate selectively in tumor cells and can serve as targeted treatment agents. While promising results have been observed in clinical trials, consistent success of therapy remains elusive. The dynamics of virus spread through tumor cell populations has been studied both experimentally and computationally. However, a basic understanding of the principles underlying virus spread in spatially structured target cell populations has yet to be obtained. This paper studies such dynamics, using a newly constructed recombinant adenovirus type-5 (Ad5) that expresses enhanced jellyfish green fluorescent protein (EGFP), AdEGFPuci, and grows on human 293 embryonic kidney epithelial cells, allowing us to track cell numbers and spatial patterns over time. The cells are arranged in a two-dimensional setting and allow virus spread to occur only to target cells within the local neighborhood. Despite the simplicity of the setup, complex dynamics are observed. Experiments gave rise to three spatial patterns that we call “hollow ring structure”, “filled ring structure”, and “disperse pattern”. An agent-based, stochastic computational model is used to simulate and interpret the experiments. The model can reproduce the experimentally observed patterns, and identifies key parameters that determine which pattern of virus growth arises. The model is further used to study the long-term outcome of the dynamics for the different growth patterns, and to investigate conditions under which the virus population eliminates the target cells. We find that both the filled ring structure and disperse pattern of initial expansion are indicative of treatment failure, where target cells persist in the long run. The hollow ring structure is associated with either target cell extinction or low-level persistence, both of which can be viewed as treatment success. Interestingly, it is found that equilibrium properties of ordinary differential equations describing the dynamics in local neighborhoods in the agent-based model can predict the outcome of the spatial virus-cell dynamics, which has important practical implications. This analysis provides a first step towards understanding spatial oncolytic virus dynamics, upon which more detailed investigations and further complexity can be built.

## Author Summary

Traditional chemotherapy of cancers is characterized by strong side effects, while showing a low success rate in the long term control of tumors. Besides small molecule inhibitors, which have shown great promise, oncolytic viruses present an emerging specific treatment approach. They are engineered viruses that spread from tumor cell to tumor cell, killing them in the process. Non-tumor cells are generally not infected. While clinical trials have given rise to promising results, reliable success remains elusive. Besides experiments, computational approaches provide a valuable tool to better understand the dynamics of virus spread through a growing population or tumor cells. Combining in vitro experimental approaches with computational models, we study the principles of virus spread through a spatially structured population of cells, which is of fundamental importance to understanding virus treatment of solid tumors. We describe different growth patterns that can occur, interpret them, and explore how they relate to the ability of the virus to induce tumor regression. We further define how these spatial dynamics relate to settings where cells and viruses mix more readily, such as in many cell culture experiments that are used to evaluate candidate viruses.

## Introduction

Oncolytic viruses replicate selectively in tumor cells and have been explored as a targeted treatment approach against cancers [1], [2], [3], [4], [5], [6], [7], [8], [9], [10], [11], [12], [13], [14], [15]. In principle an oncolytic virus will spread though the tumor cell population and lyse the infected cells, leading to eradication or control of the tumor. Because of the selectivity of such viruses for cancer cells rather than normal human cells, side effects also should be less pronounced than those associated with traditional treatments, such as chemotherapy or ionizing radiation. Oncolytic virus therapy has been explored in the context of several different virus species. While some non-human viruses display natural selectivity for cancer cells in humans [16], modern approaches use genetically engineered viruses to achieve tumor selectivity. The first engineered virus generated in the 1990s was a herpes simplex virus-1 [17]. Engineered adenoviruses have been of major interest in recent clinical trials, especially in the context of head and neck cancer [15]. Indeed the adenovirus H101(Shangahi Sunway Biotech, Shanghai, China) was approved in China for the treatment of head and neck cancer in combination with chemotherapy [18]. A variety of other virus types has also been explored [19]. However despite initial promising results and observations in the laboratory and clinic, oncolytic viruses have so far failed to demonstrate sustained and reliable treatment success [15].

Besides experimental research, mathematical and computational modeling has increasingly become a tool to study the dynamics of oncolytic viruses. Mathematical models can help us understand the emerging properties of cancer-virus interactions, to interpret experimental results, and to design new experiments. The first mathematical models of oncolytic virus therapy considered ordinary differential equations that described the basic interactions between a replicating virus and a growing population of tumor cells, and also immune responses [20], [21]. Further work extended this type of approach in a number of ways, describing different scenarios and applying models to specific virus-tumor systems [22], [23], [24], [25], [26], [27], [28], [29], [30], [31], [32], [33], [34], [35]. One of the assumptions that is implicit in such modeling approaches is that cells and viruses mix perfectly with each other (mass action). While this might hold true in the context of some in vitro experiments, and while this might be a reasonable approximation of the dynamics occurring in some non-solid tumors, the majority of tumors have intricate spatial structures where cells and viruses do not mix well, but where interactions are limited to local neighborhoods. Hence, to gain a better understanding about the dynamics of oncolytic viruses, spatially explicit models are required. While some spatial modeling studies have been performed and have given rise to interesting results [30], [36], [37], [38], they commonly include, in addition to basic spatial dynamics, one or more additional assumptions that introduce further complexity.

We still do not, however, have a good understanding of the basic principles that govern spatially restricted virus spread through a population of target cells, what outcomes can be expected, and what determines those outcomes. Obtaining such basic knowledge is a necessary foundation for building predictive models of virus therapy. This knowledge can be used as a basis for examining the effects of further biological complexities on the outcome of virus therapy, such as immune responses, tumor-microenvironment interactions, cellular heterogeneity, cell-cell interactions, among others. Therefore, the aim of this paper is to study the basic dynamics of virus spread through a spatially arranged population of growing cells in a simple setting. To achieve this, we have constructed an in vitro experimental system in which a fluorescent labeled virus spreads through a target cell population in a two-dimensional geometry, such that an infected source cell can transfer the virus only to target cells in the direct neighborhood. Besides quantifying the number of infected cells, this also allows us to track the emerging spatial patterns over time. We found three distinct patterns of virus spread and determined the frequency of their occurrence. An agent-based model was used to simulate these experiments and to interpret the data. The model can qualitatively reproduce the experimental observations and suggests key parameters that determine the different growth patterns. Using this model, we explore the implications of the observed growth patterns for the long-term outcome of the dynamics, and obtain insights about the conditions required for the virus to drive the target cell population extinct in this setting. This is a first step towards understanding the basic principles of virus spread and the correlates of successful virotherapy in spatially structured cell populations, and provides a basis for more detailed explorations and for the incorporation of other complexities that are relevant for virus-tumor dynamics in vivo.

## Results

### Experimentally observed patterns of virus spread

**Figure 1:**
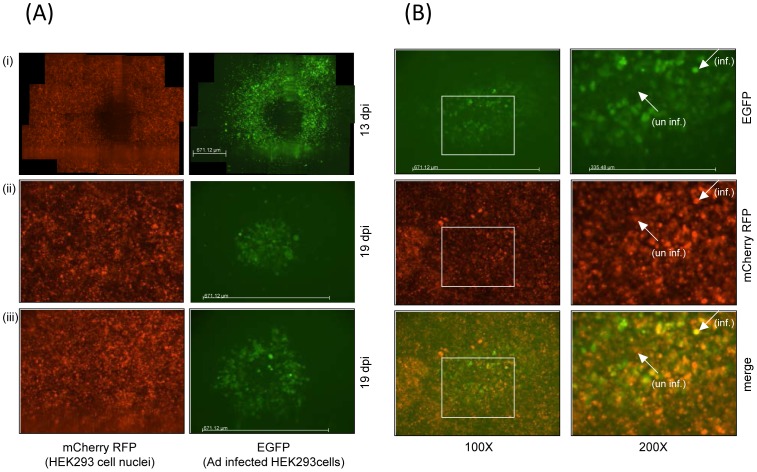
Observed patterns of AdEGFPuci infection in HEK293-mCherry cells. (A) HEK293-mCherry cells were infected at an MOI≪1 and tracked every 24 h beginning at 5 days post-infection when initial spread of infection had occurred. Three representative patterns of AdEGFPuci infection were observed after at least 13 days post infection (pattern (i)) or as long as 19 dpi (patterns (ii) and (iii)) as shown in the micrographs (100×). The left panels represent the detection of HEK293-mCherry cell nuclei in the culture (mCherry RFP), whereas the right panels depict the identical field of view of HEK293-mCherry cells viewed for green fluorescence to detect cells infected with AdEGFPuci (EGFP). (B) Limited pattern (iii) of HEK293-mCherry cells infected by AdEGFPuci. The top panels depict AdEGFPuci infected cells (EGFP-positive), the middle panels depict all the HEK293-mCherry cell nuclei in the culture (mCherry RFP), and the bottom panels is the merge of those panels, illustrating infected vs. uninfected cells. The panels on the left are micrographs taken at 100× magnification; the right panels encompass the boxed area of the left images at 200× magnification. The arrows in the right panels point to an AdEGFPuci infected cell (inf.) and an uninfected AdEGFPuci cell (un inf.) within the center of the virus infected region of HEK293-mCherry cells at 19 dpi. The scale bar in both Figure 1A and 1B for the 100× magnifications represents 671.12 uM, the 200× image scale bar is 335.48 uM. The scale bar in the 100× magnification defines the region of each micrograph needed to capture the area of the infected cells of an individual infection (compare Figure 1A pattern (i) with patterns (ii) and (iii)).

In order to examine spatial virus spread in a relatively simple setting, we constructed a recombinant adenovirus type-5 (Ad5) that expresses enhanced jellyfish green fluorescent protein (EGFP), AdEGFPuci, and grows on human 293 embryonic kidney epithelial (293) cells [39]. The experiment was set up such that cells are arranged in a two-dimensional layer, and virus spread is most likely to occur to neighboring cells. An agar overlay prevents long-range spread of the virus away from infected cells in the culture medium. This set-up allows us to not only quantify the number of infected cells over time, but also the spatial patterns of infected cells that are formed as the virus population expands. In addition, we used fluorescent markers to visualize the spatial distribution of all cells (infected and uninfected) by generating HEK293-H2BmCherry cells, that stably express the core nuclear histone protein H2B fused to mCherry (a highly photostable, monomeric red fluorescent protein (RFP)) [40]. Thus, using HEK293-H2BmCherry cells allows us to visualize all the cell nuclei (i.e., intact cells) in any particular culture. The culture was infected at a very low multiplicity of infection (MOI), such that any area of infection resulted from a single “founder” infected cell. Each culture contained several such founder cells that were sufficiently separated from each other, allowing us to track multiple growth foci across the dish. Details of the experimental procedures are given in the Methods section. The earliest stages of virus growth starting from a single founder infected cell were characterized in detail in a separate study [39]. This gave rise to the interesting observation that while virus extinction was a likely event as long as the number of infected cells in a given area was less than three, spreading virus growth was always observed once the number of infected cells reached three or higher. In the current study, we followed the growth of such spreading infections and characterized the consequent growth patterns. We observe three basic patterns of virus spread, which interestingly occur under identical experimental conditions and even within the same culture. They are shown in Figure 1A and described as follows. (i) In the first pattern, the virus infection spreads rapidly outwards as a ring, leaving no cells behind in the core of the ring (Figure 1A, pattern (i)). This classic plaque pattern is observed in virus growth experiments. We call this the “hollow ring” structure. In the second and third patters there is viral spread, but it is limited. (ii) In the second case, a “disperse” growth pattern is observed, where the virus population expands as a mixed cluster of infected and uninfected cells (Figure 1A, pattern (ii)). Finally, the virus population expands as a thinner ring, but in contrast to the first case, uninfected cells are left behind in the core of the ring (Figure 1A, pattern (iii)). We call this the “filled ring” structure. A limited growth pattern is magnified in Figure 1B, in which uninfected cells are visible within the center of the virus infected population. In the top right panel of Figure 1B, an AdEGFPuci infected (fluorescent) cell is indicated (arrow, inf.), whereas an uninfected cell in the center of the spreading infection does not fluoresce green (arrow, un inf.). The same cells are indicated in the middle right panel of Figure 1B, showing red fluorescence. In the bottom left panel of Figure 1B, images of the top and middle panels are merged; infected cell (arrow, inf.) fluoresces yellow, while the uninfected cell, (arrow, un inf.) remains red. As mentioned the area over which the infection spread remained limited in patterns (ii) and (iii) and persisted throughout the infection (through 19 dpi). In contrast, in pattern (i) the ring of infected cells continued to spread outward as long as there was space; cell clearing in the center of the plaque was apparent at 13 dpi, as shown in Figure 1A. Similar patterns of spreading infection were also seen in Ad293 cells, a HEK293 cell derivative optimized for adenovirus plaque assays. Overall, among 436 scored growth foci, the hollow ring structure was found in 45%, and the limited patterns in 55% of cases.

In the following sections, we simulate these experiments with a computational model. We examine conditions for the formation of different patterns and examine implications of the different growth patterns for the ability of the virus to eradicate the target cell population.

### An agent-based model of the experiments

Our in silico studies of the interactions between oncolytic viruses and cancerous cells rely on the agent-based modeling technique, where each cell is represented as an “agent” occupying a certain position on a grid, and interacting with other cells according to some (probabilistic) rules. Our modeling approach is spatial, that is, it takes into account the spatial distribution of the uninfected and infected cells

The model, based on previous work [41], describes target cell-virus dynamics on a two dimensional grid that contains *N×N* spots. Each spot is either occupied by a cell (infected or uninfected), or it is empty. We model the development of the populations in discrete time. Given the state of the system at time *t*, a set of rules is applied to each spot, and this gives rise to the state of the system at time *t+1*. At each time step, the grid is randomly sampled *N^2^* times. If the chosen spot is occupied by an uninfected cell, it can die with a probability *D*, leaving the spot empty. Alternatively, the cell can reproduce with a probability *R*, and a destination spot is randomly chosen for the offspring from the set of eight nearest neighboring spots. If the destination spot is empty, the offspring is placed there, otherwise, no reproduction occurs. If the chosen spot contains an infected cell, it can die with a probability *A*, or attempt to transmit the virus with a probability *B*. A destination spot is chosen randomly from the eight nearest neighbors, and infection only proceeds when a susceptible cell is present. Infected cells do not reproduce since adenoviruses lock the cell in the S-phase for replication, thus preventing further divisions [34]. The particular formulation of the infection process used in this paper corresponds to virus transmission that is not coupled with cell death, but simulations indicate that transmission coupled to cell death does not qualitatively alter the results reported here. For reference, parameters and their meaning are summarized in Table 1.

**Table 1:** Parameters of the model.

Parameter	Meaning
R	Division probability of uninfected cells
D	Death probability of uninfected cells
B	Probability for an infected cell to transmit the virus
A	Death probability of infected cells

### Initial virus growth patterns

**Figure 2:**
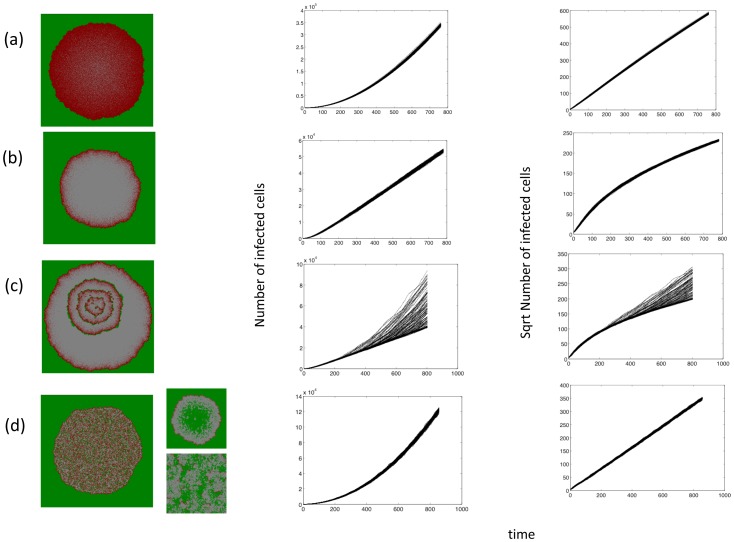
Initial virus growth dynamics in the agent-based model. Each row represents one case, characterized by a certain parameter combination. The left graph shows the spatial pattern. Green indicates uninfected cells, red infected cells, and grey empty spots. The middle graph shows the number of infected cells over time. The different lines represents 100 different instances of the simulation with the same parameter combination. The right graph shows the square root of the number of infected cells over time, again showing lines for 100 different runs. If this graph is linear, we observe quadratic or “surface” growth. Two basic types of initial growth are observed. Cases (a) and (b) show the formation of a ring structure, characterized by an initial phase of quadratic growth, and a subsequent phase of linear growth. In (a) the death rate of infected cells is lower, thus requiring a longer period of time until a hollow ring is formed. Hence, the duration of the quadratic growth phase is relatively long, almost the entire duration of the simulation presented here. Eventually, however, it will transition to linear growth, as exhibited in case (b), characterized by a faster death rate of infected cells. Case (d) shows a disperse growth law. No ring structure is formed. The virus population leaves behind susceptible cells, thus leading to a mixed pattern and quadratic growth. The small graphs depict a variation of this outcome which occurs if the viral spread rate is at the low end of the spectrum. Due to a higher number of target cells at the edge of the infection area, significant number of infected cells are only found there, and we observe a ring-like structure with a mass of uninfected cells in the center (upper small graph). Over time, the dynamics develop into a mixed pattern with low levels of infected cells (lower small graph), or the infection goes extinct. Case (c) lies in between the hollow ring and the mixed pattern. Initially a ring structure is formed, resulting first in quadratic, then in linear growth. However, on rare occasions uninfected cells remain in the wake of the expanding virus population, thus leading to concentric rings. While this does not happen in all instances of the simulations, when it does occur, the growth law transitions back from linear to quadratic. Parameter values were chosen as follows. (a) *R = 0.5*; *D = 0*; *B = 0.6*; *A = 0.601*. (b) *R = 0.5*; *D = 0*; *B = 0.6*; *A = 0.62*. (c) *R = 0.5*; *D = 0*; *B = 0.6*; *A = 0.628*. (d) *R = 0.5*; *D = 0*; *B = 0.6*; *A = 0.7*. The small graphs in (d) are characterized by *R = 0.04*, leading to fewer target cells in the area of infection and thus to slower viral spread.

In this section, we explore the initial virus growth patterns. As starting conditions we assume that the grid is filled with uninfected cells and that a relatively small square of infected cells (30×30 spots) is placed in the middle of the grid (which overall contains 300×300 spots). The emerging growth pattern depends on parameters that influence the rate of virus spread, in particular the probability for an infected cell to die, *A*, and the probability for an infected cell to transmit the virus, *B*. The patterns that we observe are presented in Figure 2.

In Figures 2a and b, the infected cell population expands as a ring or wave that leaves no cell behind in its core. The two pictures differ in the death probability of infected cells. In Figure 2a, the probability for infected cells to die is relatively low such that during the time frame of the simulation a hollow ring has not yet formed and the infected cell population expands as a relatively solid mass. In Figure 2b, the death probability of infected cells is higher such that during the time frame of the simulation a hollow ring has formed. In Text S1 we derive an approximate growth law for these scenarios. The total number of cells is proportional to 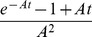, such that for short time-scales (or smaller death rates) the growth is quadratic in time, and for longer times scales (or larger death rates) it is linear in time. This is exactly what is observed. Figure 2a, characterized by smaller values of A, shows a growth law of the infected cell population that is close to quadratic. In Figure 2b, where the death rate is larger, the infected cell population grows linearly once the hollow ring is present. Note that these two scenarios are identical in principle because in Figure 2a, the formation of the hollow ring requires more time (and a larger grid). The higher the death rate of infected cells, the faster the ring is formed, and the faster the growth law changes from square to linear. Lowering the rate of virus spread (decreasing the value of *B* and increasing the value of *A*) gives rise to patterns of a different nature.

In Figure 2c, uninfected cells are left behind in the core of the expanding ring. When they grow and become infected by virus, a coupled expanding ring of uninfected and infected cells forms. This can occur repeatedly, giving rise to concentric rings. The persistence of cells in the core of the ring is probabilistic in nature, and that is reflected in the growth laws that are observed in multiple runs of the simulation. In cases where uninfected cells are not left behind inside the ring, the infected cell population grows linearly. When concentric rings do occur, the growth becomes quadratic.

Finally, no expanding ring structure is formed in Figure 2d because the viral spread kinetics are even slower. Instead, the area of virus growth is characterized by a mix of infected and uninfected cells that expands over time. In this case, quadratic growth of infected cells is observed (see Text S1). Note that if the viral spread kinetics are in the lower end of this spectrum, it is possible to observe a variation of this pattern, shown in the inset of Figure 2d: While the spreading infection leaves uninfected cells behind, the viral spread kinetics are too low to maintain significant numbers of infected cells throughout this area. Most of the infected cells will be at the outer edge of the infection due to a higher density of target cells. In this case, a relatively thin, ring-like structure can be formed, with a large area of uninfected cells remaining in its core. This pattern, however, is temporary. With time, one of two scenarios can be observed. A mixed pattern can be generated, characterized by a large number of uninfected cells and a low number of infected cells, because the virus eventually spreads to the remaining susceptible cells. Alternatively, there is a chance that the virus population goes extinct due to the slow rate of spread. Long term outcomes are discussed further below.

Figures 2a and b qualitatively correspond to experimental pattern (i), the hollow ring structure. Figure 2d corresponds to experimental pattern (ii), the disperse growth structure. The pattern shown in the inset of Figure 2d likely corresponds to experimental pattern (iii), where a limited ring with mainly uninfected cells in its core temporarily forms before either developing into a mixed pattern or resulting in virus extinction. According to this interpretation, the experimentally observed patterns (ii) and (iii) are variations of the same theme. The concentric rings observed in the model simulations are not found in the experimental data. This is not surprising because they only occur in relatively narrow parameter regions in the model.

**Figure 3:**
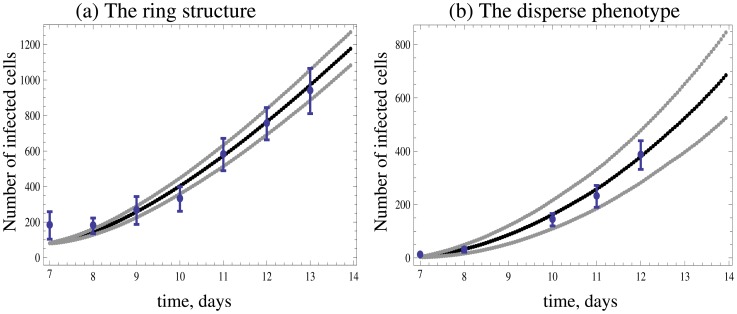
Two different experimentally observed time series of adenovirus (AdEGFPuci) growth on human 293 embryonic kidney epithelial cells, arranged in a two dimensional layer. The number of cells was determined by measuring the fluorescent area of the infected cell population, divided by the fluorescent area of individual infected cells, using Photoshop (see Methods as well as Text S1). The graph is based on a single experimental run. The area however, was measured independently four times, giving rise to the plotted error bars. The black middle line through the data represents the time series predicted by the agent-based model, using a parameter combination that was obtained by a least squares fitting procedure. Since the model is stochastic, the predicted time series represents the average over 1000 instances of the simulation. The upper and lower lines show the standard deviations added to and subtracted from the average. (a) This experiment shows virus growth characterized by the formation of a ring structure. Consequently there is a relatively short phase of quadratic growth, followed by a transition to linear growth. (b) This experiment shows disperse virus growth characterized by quadratic growth throughout time. The corresponding observed and predicted spatial patterns are shown in Figures 4 and 5. Values for parameters R, B, and A were obtained from the fitting procedures and are given as follows: (a) *R = 0.18*, *B = 0.26*, *A = 1.85×10^−2^*. (b) *R = 0.19*, *B = 0.52*, *A = 0.12*. The parameter *D* was kept constant at *D = 0*. For further details of the fit, including initial conditions and the time step of the simulation, see Text S1.

**Figure 4:**
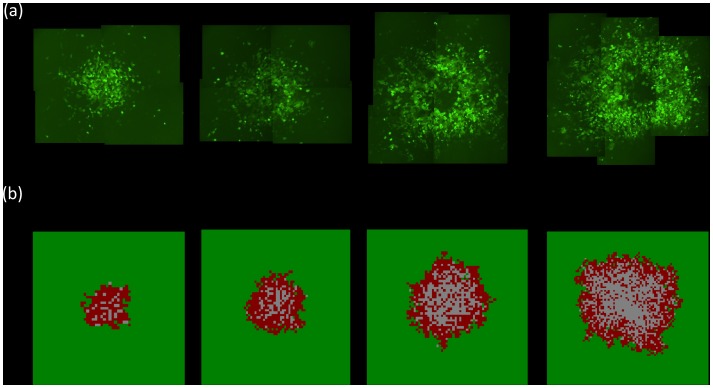
Observed (a) and predicted (b) spatial pattern of adenovirus (AdEGFPuci) growth for the experiment that exhibits a ring structure (time series given in Figure 3a). The predicted spatial pattern is the result of an individual run of the agent-based model with the parameter combination obtained from the model fitting procedure. Snapshots in time are shown, representing days 7, 9, 11, and 13 post infection. (a) The area of green fluorescence is shown, expressed by the infected cells, thus documenting the spatial spread of the virus through the population target cells arranged in a two dimensional setting. (b) In the computer simulation, green indicates infected cells, red infected cells, and grey empty spots.

**Figure 5:**
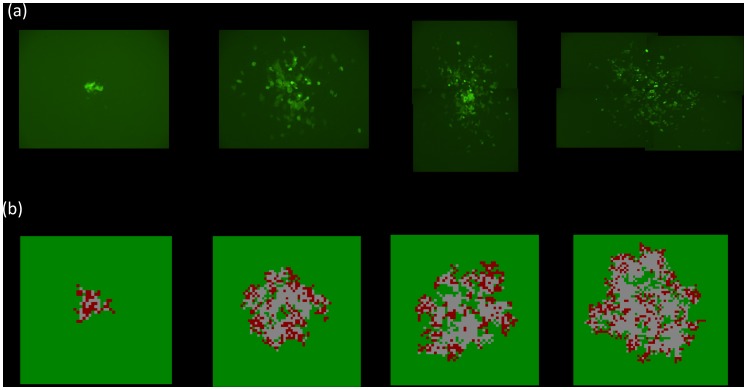
Observed (a) and predicted (b) spatial pattern of adenovirus (AdEGFPuci) growth for the experiment that exhibits a disperse growth pattern (time series given in Figure 3b). The predicted spatial pattern is the result of an individual run of the agent-based model with the parameter combination obtained from the model fitting procedure. Snapshots in time are shown, representing days 7, 10, 11, and 12 post infection. (a) The area of green fluorescence is shown, expressed by the infected cells, thus documenting the spatial spread of the virus through the population target cells arranged in a two dimensional setting. (b) In the computer simulation, green indicates infected cells, red infected cells, and grey empty spots.

In order to go beyond the qualitative comparison of model and data, we fit the model to two sets of experimental data, one showing an expanding hollow ring, and the other the disperse growth pattern. A least squares algorithm (see Text S1) was used to fit the number of infected cells over time, and a relatively good fit was obtained for both cases (Figure 3). The types of spatial patterns that emerged matched the observed ones qualitatively (Figures 4 and 5). While this procedure found best fitting parameter values, their biological meaning remains questionable, since different parameter combinations can give rise to similarly good fits. A more solid validation would require an independent estimation of parameter values, and a subsequent generation of the predicted growth patterns. Due to the complexity of the experimental observations, this is not currently possible and is discussed in detail below. The fitting procedure does, however, indicate that the model is at least consistent with experimental data.

### Growth patterns and the extinction of cells

**Figure 6:**
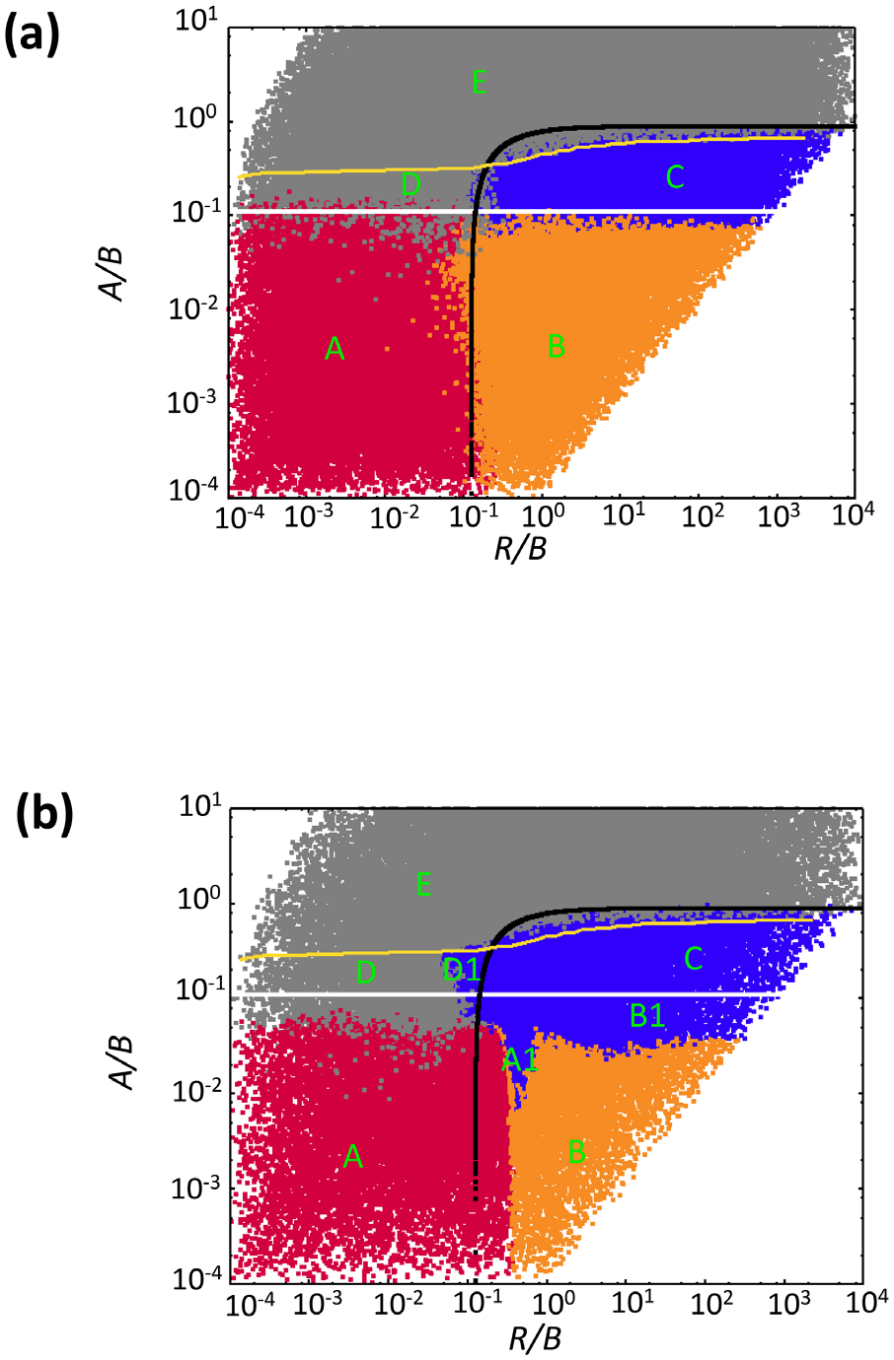
Dependence of outcomes on parameters in the agent-based model for *(a)* a relatively small 30×30 grid, and *(b)* a relatively large 300×300 grid. The plot is the result of at least 10^4^ instances of the simulations, where the log_10_ of the parameters was varied between −4 and 4. The simulations were started by placing a small number of infected cells (5×5 cells) into a larger space filled with infected cells (13×13 cells). Identical results were observed over a very large range of initial conditions (differences were only observed if the initial number of cells is such that immediate stochastic extinction is likely, which are not regions of interest with respect to our study). Blue indicates coexistence of virus and cells. Red and orange indicate extinction of the cells and thus the virus. Red is used if extinction occurs before the boundary of the system has been reached, while orange is used if extinction occurs after cells have reached the boundary of the system. Grey indicates extinction of the virus while cells persist. Above the white line and below the black line, the “local” equilibrium number of uninfected and infected cells, respectively, is greater than one. This derives from ordinary differential equations that describe the dynamics within local neighborhoods where all cells can interact with each other (see Text S1). Below the yellow line, the virus can successfully invade its target cell population. This invasion threshold was determined by numerical simulations. The capital letters indicate different spatial patterns that are described in the text and in Figure 7. In these simulations, the probability for an uninfected cell to die was kept constant at D = 0.

**Figure 7:**
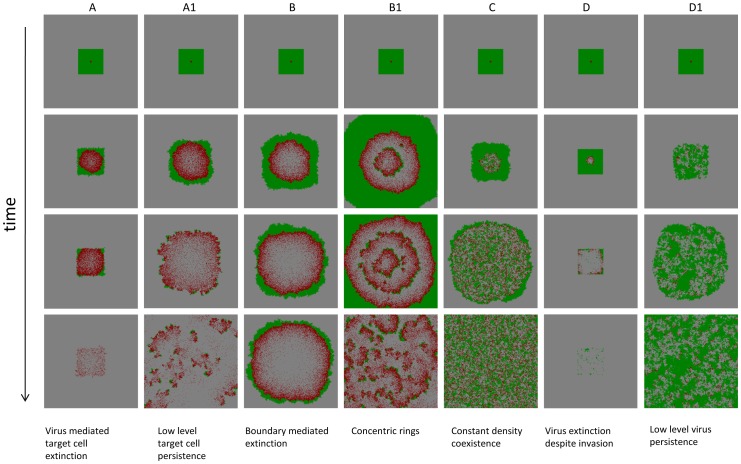
Seven spatial patterns observed in the agent-based model. For each pattern, four snapshots in time are shown. Green indicates uninfected cells, red infected cells, and grey empty patches. See corresponding capital letters in Figure 6, showing in which parameter regions the individual patterns are observed. The time series that are associated with the individual patterns are shown in Figure 8. See text for details. The simulations were run on a 300×300 grid. The simulations were started by placing a small number of infected cells (5×5 cells) into a larger space filled with infected cells (13×13 cells). Parameters were chosen as follows: (A) R = 0.013; D = 0; B = 0.14; A = 0.003; (A1) R = 0.15; D = 0; B = 0.32; A = 0.007; (B) R = 0.014; D = 0; B = 0.015; A = 0.00056; (B1) R = 0.04; D = 0; B = 0.032; A = 0.0016; (C) R =  0.014; D = 0; B = 0.032 ; A = 0.008; (D) R = 0.0002 ; D = 0; B = 0.019 ; A = 0.0032 ; (D1) R = 0.069 ; D = 0; B = 0.64 ; A = 0.18.

**Figure 8:**
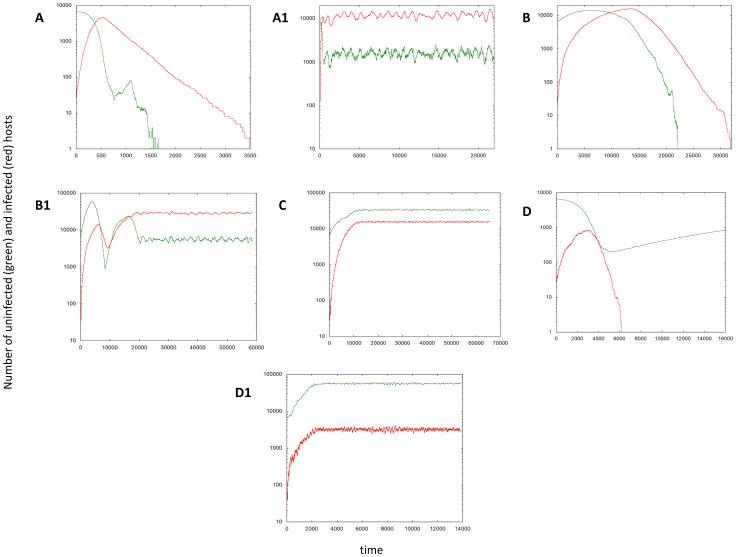
Typical time series corresponding to the spatial patterns presented in Figure 7, based on a single run of the spatial agent based model, assuming a *300×300* grid. See text for details. Parameter values and initial conditions are given in Figure 7.

Here, we explore the long term dynamics, investigating how the above described patterns play out and correlate with the overall outcome if both the uninfected and infected cell population can expand in space. We seek to define conditions under which the virus can eliminate the target cell population in this system. All simulations are started with a small number of infected cells placed in a compact vicinity into a larger space filled with uninfected cells, which is in turn embedded into an even larger “empty” space (for the exact initial conditions for particular cases, see appropriate figure legends). In contrast to the simulations reported above, here we go beyond the initial virus growth stage, and focus on time-scales where the population of target cells experiences significant changes (grows in size in the absence of infection). The outcomes of this system include extinction of the target cells and thus the virus; extinction of the virus and persistence of the target cells; coexistence of virus and target cells. The dependency of these outcomes on the parameters is shown in Figure 6, which is the result of at least 10^4^ instances of the simulation, where the log_10_ of all the parameters was varied between −4 and 4. Figures 7 and 8 show corresponding spatial and temporal patterns. We examine the outcomes first in a relatively small *30×30* grid, and subsequently in a larger, *300×300* grid.

#### Small grid

In the 30×30 grid, the following outcomes are found (Figure 6a, and Figures 7 & 8).

Two types of target cell extinction can be observed, both associated with the initial “hollow ring” structure. According to pattern **A**, ***virus-mediated target cell extinction***, both the target cell and the virus populations spread outward in space as a wave, but the virus wave overtakes the target cell wave, leading to extinction of both populations. Pattern **B**, ***boundary-mediated extinction***, represents weaker viruses compared to case A. In pattern B, the virus wave catches up with the target cell wave, leaves no uninfected cells behind in its wake, but fails to eliminate the target cell wave. Instead, the two waves travel together with the same velocity until the boundary in reached. The target cell population can escape the virus only by spreading outward. Once the boundary is reached, this is not possible anymore, explaining the extinction. Note that although real tumors are capable of breaking out of homeostatic control and spreading beyond the “carrying capacity” of their environment, boundary-mediated extinction can still take place. Genetic transformations associated with waves of clonal expansion or induction angiogenesis generally happen on longer time-scales. Therefore, it is realistic to assume the existence of some geometric constraints (even temporary). Pattern B represents the situation where extinction is a consequence of such spatial constraints.

Another type of outcome is the coexistence of infected and uninfected cells, which is shown in pattern **C**, ***constant density coexistence***. As the virus population spreads out in space, it leaves behind uninfected cells with a high probability, leading to the disperse pattern of initial expansion and the absence of any clear traveling waves. Instead, the expanding virus population leaves behind a mix of both populations, which eventually is found across the whole space and is characterized by an equilibrium density that is determined by the parameters of the system, while the populations settle around a stochastic steady state.

Finally, there are two types of virus extinction patterns. Pattern **D**, ***virus extinction despite invasion***, represents a virus extinction regime where the virus can initially invade the target cell population, but does not persist in the long term. The virus reduces the target cell population, and subsequently goes extinct. This leaves the uninfected cell population to grow unopposed. The stronger the virus (lower value of *A/B*), the less likely this is observed in this regime, because the uninfected cell population is more likely to be driven extinct before the virus population hits extinction. The second extinction pattern **E**, ***lack of invasion***, is observed when the virus population cannot invade the target cell population and goes extinct (spatial and temporal pattern not shown).

#### Analytical insights: global outcome can be predicted by the local dynamics

Although the spatial stochastic predator-prey system studied here exhibits a variety of patterns, its dynamics can be understood by studying the local interactions of the agents. The idea of a “characteristic scale” has been proposed in the literature in the context of different predator-prey models [42] where the system's behavior was found most predictable on an intermediate scale defined by the agents' motility and interactions. In [43], it was shown that in a class of systems exhibiting oscillatory dynamics, the functional forms governing the local predator-prey interactions at those characteristic scales are the same as the ones describing a perfectly mixed, mass-action system, but contain different parameters. This allowed the authors to approximate the long-term dynamics of the spatial system at large scales with a temporal predator-prey model describing local interactions. In this paper we will build on this idea, and show that the global outcomes of the spatially-distributed system can be predicted by utilizing the laws of local dynamics, see Text S1 for more details.

We start from the well-known system of ordinary differential equations that can be derived for our agent-based model if no spatial restrictions were in place, and reproduction and infection events were driven by laws of mass-action:
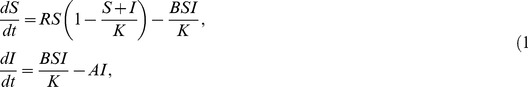
(1‐2)where the number of uninfected cells is denoted by *S*, and the number of infected cells by *I*. In these equations, *K* has the meaning of carrying capacity. This well-known modified Lotka-Volterra system [44], [45] is characterized by two equilibria: (i) The uninfected population persists at carrying capacity, while the virus population is extinct, i.e. *S^(0)^ = K*, *I^(0)^ = 0*; (ii) Alternatively, the virus establishes a successful infection, such that *S^(1)^ = AK/B*, *I^(1)^ = RK(B−A)/B(R+B)*. The latter equilibrium is stable if the basic reproductive ratio of the virus is greater than one, which is equivalent to the inequality A<B. The approach to the coexistence equilibrium can be either monotonic, or can involve damped oscillations.

While these properties of the virus-cell system are well-known, it is usually thought that the ordinary differential equations can only be applied to a well-mixed system, and fail to describe a spatially-distributed system of cells. Contrary to this, Figure 6 demonstrates that, if interpreted correctly, the above system can explain a lot of the patterns that arise in the spatial agent-based model. Let us think of the carrying capacity coefficient, K, as the size of the “local neighborhood” where cell-to-cell interactions happen in a spatial model. In our case, this neighborhood consists of K = 9 cells (a cell plus its eight nearest neighbors, the relevant characteristic scale of our spatial model). Equations (1–2) with the modified parameter K are capable of informing us of the local equilibrium density of the infected and uninfected cells, which in term is correlated with the expected long term behavior of the spatial system.

In equations (1–2), the number of uninfected cells at the equilibrium (the value *S^(1)^*) is proportional to K. In order for this equilibrium to be biologically meaningful, this value must be greater than one cell. The equation *S^(1)^ = 1* defines the white line in Figure 6. Similarly, the number of infected cells in local neighborhoods must be greater than one, which yields the black line, *I^(1)^ = 1*. We can see that the coexistence region in Figure 6a (regime C) corresponds to the parameters for which both equilibrium values are larger than one; it is enclosed by the lines *S^(1)^ = 1* and, *I^(1)^ = 1* obtained directly from the cancer-virus equations. The white line *S^(1)^ = 1* outlines the lower boundary of the coexistence region, while the black line *I^(1)^ = 1* defines the upper boundary. (A more precise definition of the upper bound of the coexistence region is given by the yellow line in Figure 6a, below which the virus is strong enough to invade the cell population).

Thus, in the spatial system, target cell extinction is observed if the local equilibrium number of uninfected cells is less than one (regions A & B, Figure 6a, below white line). Extinction of virus only is observed either following initial invasion if its local equilibrium is less than one (region D, Figure 6a, area encased by white, black and yellow lines) or if invasion is impossible (region E, Figure 6a, above yellow line). The finding that equilibrium properties of simple ODE models that describe the dynamics in a small local neighborhood can predict the outcome of the spatial system has important practical implications. Note however that this method is unable to explain all the details of the diagram in Figure 6. In particular, the proximity of the black (*I^(1)^ = 1*) line to the boundary between regions A and B is purely coincidental. The equilibrium analysis predicts extinction in regions A and B, but cannot distinguish between virus-mediated extinction (A) and boundary-mediated extinction. We will return to this aspect of the diagram armed with the tools from PDE analysis, see below.

#### Large grid

In a larger, 300×300, grid (Figure 6b, and Figures 7 & 8), the basic patterns found in a small grid are still in place, but additional complexity is observed. In the parameter space where target cell extinction happens in the smaller grid, regions of coexistence can occur. In pattern **A1**, the expanding virus wave proceeds initially as a “hollow ring” structure, catches up with the target cell wave, leaves no uninfected cells in its wake, but only partially breaks the target cell wave. The virus is not efficient enough to eliminate the target cell wave, as observed in pattern A, but still strong enough not to leave it intact, as observed in pattern B. The partially broken wave structure allows the uninfected cells to escape not only outward, but in all directions. Hence, local extinction combined with continuous target cell movement away from the virus leads to persisting moving fronts, which can go extinct and give rise to new fronts over time. Thus, more extensive population fluctuations are observed in the long run (Figure 8). This is the well-known regime of global persistence despite local extinction which is an important basis for the argument that space promotes coexistence [46]. The levels at which the uninfected cell population persists, however, are relatively low (Figure 8). A sufficiently large grid size is required to observe this behavior, such that enough space is available for the moving target cell fronts to persist. We refer to pattern A1 as ***low-level target cell persistence***. Region **B1** shows a different reason for target cell persistence at low levels, a pattern we call ***concentric rings***, which corresponds to the concentric ring pattern of initial virus spread described earlier. When the virus wave expands, the probability to leave behind uninfected cells is proportional to the local equilibrium number of uninfected cells. In the region where this equilibrium number is just slightly below one, this does not occur often enough to be observed on a small grid. On a larger grid, however, it can be observed. These infrequent events lead to renewed target cell growth, followed by virus growth, and a new wave structure is formed. This can lead to the occurrence of concentric expanding rings. With time, stochasticity breaks the ring structure, leading to traveling fronts that eventually go extinct, but occasionally leave behind uninfected cells to form new fronts, thus persisting in the long term. Consequently, populations show more extensive fluctuations around characteristic steady state values (Figure 8). For lower values of *A/B*, the local equilibrium number of uninfected cells becomes too low for this to be observed in the grid size under consideration. Finally, in region **D1**, ***low-level virus persistence***, global persistence of the virus despite local extinction is observed, leading to relatively strong population fluctuations (Figure 8). While the virus invades the target cell population, it converges to its local equilibrium value that is less than one. However, movement through space before extinction occurs allows coexistence if the grid is sufficiently large. For lower values of *R/B*, the local equilibrium number of infected cells is too low to observe this outcome even in the context of the larger grid.

This analysis shows that increasing the grid size allows more complex outcomes to occur and increases the parameter region in which the cell populations persist. The additional patterns that emerge in larger grids are variations of those found in the smaller grid and involve non-equilibrium persistence, where extinction occurs locally, but movement through space allows cells to temporarily avoid extinction. These dynamics are well documented in the ecological literature [46]. Besides allowing cells to move through space, a larger grid size also increases the chances that certain rare events can occur. For example, boundary-mediated extinction (pattern B, Figure 6) is less likely to occur in large grids. The larger the grid the higher the probability that uninfected cells are left in the core of the ring before the uninfected cell population has moved to the boundary and is eliminated by the virus. All these non-equilibrium persistence outcomes in larger grids, however, are characterized by persistence of the cells at very low levels, which can be considered controlled persistence and does not involve uncontrolled cellular growth. Therefore, the outcome can still be predicted by the “local mass action equilibrium values” discussed above: if the local equilibrium of uninfected cells, predicted by the ODEs, is less than one, we can expect either extinction or controlled persistence. If the local equilibrium of uninfected cells is greater than one, we can expect to see uncontrolled cellular growth. The lower the local equilibrium of uninfected cells the less likely controlled persistence occurs and the more likely extinction is observed. However, this could not be demonstrated systematically for larger grids due to the extensive computational costs involved.

The long term outcomes shown in Figure 7 are obviously related to the initial growth patterns described in Figures 1 and 2. Patterns A, A1 and B in Figure 7 arise out of the hollow-ring structure. Pattern B1 in Figure 7 emerges from the concentric ring structure. Patterns C, D, and D1 are consequences of the disperse growth pattern/filled ring structure, which for faster viral spread rates typically leads to coexistence of the virus and cell populations, while extinction of the virus population can be observed for smaller virus spread rates.

#### Analytical insights: a metapopulation and a PDE description

To gain further understanding of the complex dynamics exhibited by the spatial stochastic agent-based simulations, we have implemented several analytical tools. While some of them such as the pair approximation method [41], [47], [48], [49], [50] did not prove particularly useful (see Text S1), other methodologies provided interesting insights which we briefly describe below.

A metapopulation approach is a modeling technique which allows us to study dynamics in a spatial setting. The model consists of a collection of *n* local patches. Within individual patches, local dynamics occur according to mass-action rules. The patches are coupled to each other by populations migrating between them. Here, we consider a one-dimensional stochastic metapopulation model where populations in a given patch can only migrate to the nearest patches. Local dynamics are described by a stochastic version of predator-prey interactions of equations (1–2) (see Text S1 for details). Uninfected and infected cells migrate to the two nearest patches with rates *m_S_* and *m_I_*, respectively. The inclusion of migration is an assumption which we only made in the metapopulation model, and not in the agent-based model. In the agent-based model, populations move through space by individuals placing their offspring to the nearest neighboring spots. This, however, is not possible in the metapopulation setting. We assume that the migration rates are equal to each other, *m_S_* = *m_I_*. In this case, no additional asymmetries are introduced that are not found in the agent-based model. This difference in information transmission through space (via divisions and infections in the agent-based model, and via explicit migration between patches in the metapopulation model) is the reason why the local rates of division, infection, and death in the metapopulation model are not the same as the agent-based model parameters *R*, *B*, and *A*. In order to distinguish the rates in the two models, we will denote the rates of division, infection and death in the metapopulation model by symbols *r*, 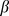, and *a*. The carrying capacity of a local patch is denoted by *k*.

Restricting the model to a one-dimensional setting greatly speeds up computing times while allowing us to verify our main results in the context of this model. A full analysis of the stochastic metapopulation dynamics, including a two-dimensional setting, will be provided in a subsequent paper.

**Figure 9:**
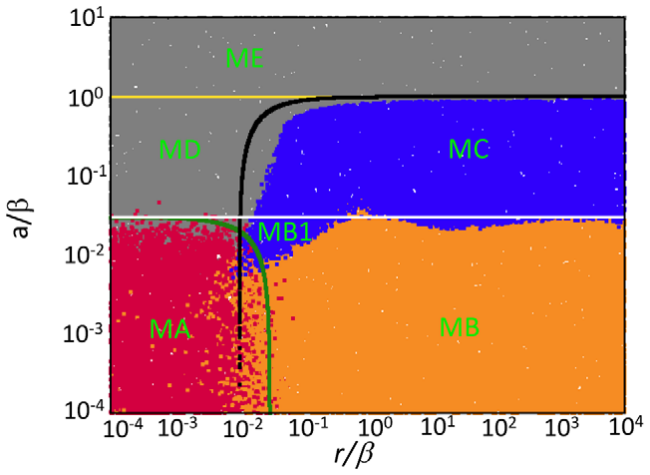
The phase diagram of the metapopulation model showing different outcomes of the dynamics depending on the parameters. The plot is a result of at least 10^4^ instances of the simulations, where log_10_ of the parameters was varied between *−4* and *4*. The metapopulation consisted of 100 local populations or spots, each characterized by a carrying capacity *k = 100*. In the middle spot, the simulation was started with 30 infected and 70 uninfected cells. In the subsequent five spots to the left and to the right of the middle spot, the uninfected cell population was at carrying capacity, without the presence of infected cells. The rest of the spots were initially empty. Blue indicates coexistence of virus and cells. Red and orange indicate extinction of the cells and the virus. Grey indicates extinction of the virus while cells persist. Above the white line and below the black line, the local equilibrium number of uninfected and infected cells, respectively, is greater than one. Below the yellow line, the virus can successfully invade its target cell population, derived from the basic reproductive ratio of the virus. See text for more details and a description of all the outcomes.

As initial conditions we assume that in a subset of adjacent local patches in the middle of the metapopulation, target cells are present at their infection-free equilibrium levels. The rest of the patches are initially empty. A small amount of infected cells are placed into the middle patch, and the infection is allowed to spread from there (See appropriate figures for exact initial conditions). Figure 9 shows the diagram of various outcomes observed in this model, and it is remarkably similar to the diagrams produced by the agent-based models. The same basic outcomes are observed: extinction of the target cells and consequently the infected cells; extinction of the infection and persistence of the target cells; coexistence of infected and uninfected cells. As before, there are two modes of extinction: in scenario **MA**, the virus is sufficiently strong, such that when it expands as a wave, it leaves no target cells behind in its wake, and overtakes the expanding target cell wave, leading to extinction of both populations. In scenario **MB**, a weaker virus can still expand as a wave, leave no susceptible cells in its wake, and catch up with the target cell wave. However, it fails to destroy the target cell wave. Instead, the two waves travel together with the same velocity until the boundary is reached, at which point extinction occurs. In region **MB1**, the same basic dynamics occur as in region MB. However, the traveling waves can split and travel independently in different directions, thus avoiding extinction when the boundary is reached (i.e. before a wave hits the boundary and goes extinct, it gives rise to another waves that travels independently). Thus, while local extinction occurs, the populations are maintained globally through the traveling waves. In region **MC** we observe “true coexistence”, where populations eventually persist locally across most of the patches. Finally, virus extinction can be observed either following initial invasion (region **MD**) or if the local basic reproductive ratio of the virus is less than one resulting in failure to invade (region **ME**).

In full analogy with the agent-based simulations, the results of metapopulation simulations can be described by looking at the equilibrium values for infected and target cells in local patches. We solve equations (1–2) where the carrying capacity is given by that of a local patch, and plot the lines corresponding to *S^(1)^ = 1* and *I^(1)^ = 1*, see the white and the black lines in Figure 9 respectively. Like in the case of the agent-based model, the local equilibrium theory is unable to predict the boundary between regions A and B. To bridge this gap of understanding, we turn to a different set of tools.

The advantage of a metapopulation model is that partial differential equation (PDE) techniques can be used to approximate some aspects of the dynamics, and to obtain further analytical insights into the behavior of the system. Although PDEs of reaction-diffusion type fail to describe stochastic phenomena such as extinction, they provide valid information in the regimes where one observes wave propagation. One can model the spatial dynamics by the following equations,
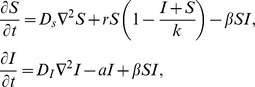
where parameters *r*, 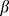, *a*, and *k* are the rates of division, infection and death, and the local carrying capacity. They are the same as in the metapopulation model. The first term in both equations describes diffusion of the target and infected cells respectively. The diffusion coefficients are related to the migration rates as 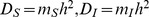, and *h* is the scaling factor describing the spacing between individual patches. Let us assume that initially we have a region that contains infection (nonzero *I*) immersed in a larger region where target cells are at their carrying capacity with no infected cells (*S = k*, *I = 0*), which in turn is immersed in an infinitely-large domain with *S = I = 0*. Both the target cells and the infected cells will spread outward. Initially, the front of the *S*-wave will be ahead of the front of the *I*-wave. The speed of propagation of the two waves, *v_S_* and *v_I_*, can be calculated by standard methods described e.g. in [51]. We have the following estimates:
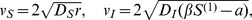
where *S^(1)^* is the equilibrium value of *S*. The validity of these estimates has to be questioned as it is well-known that noise can significantly modify the propagation velocity [52]. Comparison with numerical results shows that despite the stochastic nature of the underlying metapopulation system, the wave velocities calculated from the deterministic PDEs are in a good agreement with computational results for the propagation speed.

Using the above estimates, we can identify the conditions under which the wave of infected cells destroys the wave of target cells, leading to virus-mediated extinction (regime MA), as opposed to the scenario where the two waves travel together (boundary-mediated extinction, region MB). If the wave of infected cells travels faster than the wave of target cells (*v_S_>v_I_* ), the *I*-wave of infected cells will eventually catch up with the *S*-wave. Once the *I*-wave reaches the *S*-wave, the infected cells start lowering the level of the target cells at the front. As the level of target cells lowers, the velocity of the *I*-wave decreases (as follows from the formula for *v_I_* above). Therefore, as the region of infected cells expands toward the boundaries of the growing target cell region, the density of target cells decreases, and the wave of infected cells gradually slows down, until the speed of the *I*-wave becomes equal to the speed of the *S*-wave. From the equation *v_S_* = *v_I_* we obtain a measure of the target cell density of the expanding wave (where we set *D_s_ = D_I_*):
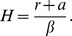



Note that in this formula we set *D_s_ = D_I_* to match our symmetric model; the more general quantity for the nonequal rates is given by 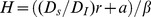. The knowledge of *H* helps us estimate the fate of the wave of target cells after the infection reaches it. Will the virus destroy the target cells, or will the two waves continue to propagate together until the boundary is reached? In Figure 9, the green line corresponds to a constant value H = 1, such that to the left of this line, virus-mediated extinction is more likely (regime MA), and to the right of this line, we expect boundary-mediated extinction regime (MB).

The above considerations help explain the qualitative shape of the phase diagram for the agent-based model. While establishing the exact match between the agent-based model and the metapopulation model goes beyond the scope of this paper, the above arguments demonstrate that

1. the patterns observed hold both in agent-based and in metapopulation models,
2. the local equilibrium argument allows us to map out many important features of the system's phase space in both models (which is especially surprising in the case of the agent-based model),
3. the PDE approach allows us to explain (and predict quantitatively in the case of metapopulation modeling) the front-propagation aspects of the dynamics.

## Discussion

We used a newly designed virus, AdEGFPuci, replicating on 293 embrionic kidney epithelial cells in order to study the spatial dynamics of virus growth in a two-dimensional setting in which a source cell transmits the virus to target cells in the immediate vicinity. We found that when the virus is placed into the midst of a target cell population, the initial growth pattern can be divided into two categories. Either the infected cells expand as a wave and eventually form a hollow ring, or the hollow ring is not formed and we observe cells remaining in the core of the ring which can lead to a disperse growth pattern. An agent-based model was used to qualitatively simulate these experiments. The model produced the same types of patterns found in the experiments and model fitting to the experimental data has shown that our description is consistent with observation. The data, together with the model, gave rise to a number of insights. When a ring is formed, the initial virus growth is quadratic and then becomes linear. In the absence of a ring, the entire virus growth is quadratic. These growth patterns are obviously related to and have implications for the dynamics of viral plaque formation, which have been previously investigated mathematically from other angles [53], [54], [55]. Initial virus growth patterns are correlated with the long-term outcomes of the dynamics. If a disperse growth structure is formed, persistence of the target cells at high levels is the only outcome, corresponding to definite treatment failure. On the other hand, the long-term outcome of the dynamics when a ring structure is formed includes extinction or low-level persistence of the target cells. In the best case scenario (which corresponds to parameter region A in our notations), the wave of infected cells catches up with the spreading target cells and drives the cell population extinct, which is the most desirable treatment outcome. Low-level persistence occurs when the virus wave fails to overtake and eliminate the target cell wave, resulting in a non-equilibrium persistence situation that requires larger grids in the model. These outcomes are likely to be relevant to tumors since the number of cells in a tumor is significantly larger than the number of cells present in any of our simulations. Cell persistence in this case, however, occurs at relatively low levels and this can be thought of as controlled persistence. Since a tumor is less likely to cause morbidity or mortality in such a state, this outcome can be considered as partially successful treatment.

An important theoretical finding is that the outcome of the spatial system (extinction versus persistence) can be predicted well from the local dynamics, characterized by the interactions among neighboring cells. In our model, this is given by the neighborhood of 3×3 spots. This result suggests that ordinary differential equations, which describe mass action dynamics, can be a valid approach to study the correlates of successful virus therapy even for tumors that exhibit spatial structure. The results of such approaches have to be correctly interpreted to include the notion of local neighborhoods. If interpreted correctly, insights gained from such previous modeling studies are applicable to the treatment of spatial tumors. In addition, this result tells us that simple in vitro experiments (where viruses and cells mix well) can be used to compare the basic replication and spread efficiencies of different viruses, and that this directly correlates with their ability to fight a spatially structured tumor. Of course, the correlates of successful treatment derived from such simple modeling studies are qualitative in nature. That is, it is for example possible to predict the effect of increasing a certain parameter, such as the death rate of infected cells, on the outcome of treatment. However, predicting whether or not virus treatment will result in the eradication/control of tumor cells is a very difficult problem, and so far no modeling approach exists that is suited to perform this task. Not only do we lack sufficient biological information, the same biological processes can be described mathematically in different ways, adding uncertainty to these models. As a next step it will be important to examine whether the global spatial dynamics can still be predicted from local mass-action dynamics in models when further complexity is introduced, including three-dimensional spatial structures, immune responses, physical barriers to spread, or cell populations with differential susceptibility to infection. If our result holds, then relatively simple ordinary differential equation models can be used to guide the exploration of other, more complex modeling approaches, as well as the design of experiments aimed at evaluating candidate viruses.

To put this work in the context of the existing literature, we note that experimental research has made much progress in the construction of viruses, in elucidating the molecular biology of virus replication in tumor cells, and in investigating clinical trials and correlates of success for several different types of candidate viruses and different tumors. Yet, the complex and multi-factorial interactions between viruses, their target cells, and other relevant components make it difficult to predict the outcomes of such dynamics, an area where mathematical tools can be of great help to complement experimental analysis, interpret data, and make experimentally testable predictions. In the recent years, there have been several mathematical/computational studies which examined spatially explicit models in more or less complex settings, taking into account an array of relevant factors that might affect the *in vivo* virus spread through a tumor [30], [34]–[36]. Here, we took a step back and examined the basic spatial dynamics between a virus and its susceptible tumor cells, ignoring more complex factors such as the immune system, tumor vascularization, or physical barriers to virus spread within tumors. In principle, the simpler setting allows for more solid experimental testing and validation of models. As it turns out, the dynamics in such a simplified setting already exhibit very complex behavior, and it is imperative to gain a thorough understanding of such a basic system before venturing on towards more comprehensive scenarios. This paper provides a first step to such an understanding.

Due to the complexity of the experimentally observed dynamics, further questions remain that are subject to ongoing investigation. The most puzzling observation was that identical experimental conditions, using the same virus-target cell system, gave rise to different patterns of virus growth. This indicates the existence of so far unidentified factors that influence virus spread in this *in vitro* system. It is possible that initial events, stochastic in nature, might determine the remaining fate of the virus population. One hypothesis is that infection of cells triggers the production of anti-viral factors by the infected cell, which could induce an anti-viral state in neighboring cells. An example of such a factor could be interferons. Indeed adenovirus infection has been reported to induce interferon beta production [56], and 293 cells respond to interferon beta by activation of interferon-responsive genes (System Biosciences. Interferon Response Detection Kit – User Manual. Mountain View, CA). Cells in the anti-viral state can have both a reduced susceptibility to infection and/or an increased death rate through the induction of apoptosis if they do become infected [57]. In such a setting, it is possible that a race occurs between the spread of the virus and the anti-viral factors to neighboring cells. The population that initially gains the upper hand in this race could determine the emerging pattern of virus growth. The hollow ring structure could be formed if the virus out-runs the anti-viral factors, and the disperse growth pattern could be observed if the virus population fails to do so. While parameter estimates from the model fit to the data remain inconclusive, the fit accounted for the disperse pattern through an increased death rate of infected cells, which might suggest that apoptosis significantly contributes to the anti-viral state. This concept could also explain the observation that the area of infection remains limited when the disperse growth pattern is found in the experiments. In our model, which does not take into account such anti-viral factors, the infection area continues to grow, albeit slowly. Interferons have been shown to influence the development of plaques before in the context of herpes simplex virus [58], and the dynamics have been examined with a similar modeling approach [59]. In this study, however, different dynamics and outcomes were observed.

The finding that different patterns can form in identical experimental conditions also blocks our ability to turn our model into a truly predictive one. This would require parameters to be measured independently in the experimental system, and to demonstrate that these parameters yield a satisfactory fit of the model to the data. However, the dynamics are most likely characterized by different parameter combinations in the context of the different observed patterns. Before we fully understand the reasons for the various patterns, parameterization of the model remains an impossible task. Nevertheless, the model analysis presented here does highlight the parameters that determine the different outcomes, which guides our search for the responsible mechanisms. Identifying these mechanisms still requires extensive work which goes beyond the scope of the current paper.

We conclude that the dynamics of virus spread in the simplest spatial setting can be very complicated. We interpreted these dynamics with a computational model, and shed light onto the meaning of the different spatial patterns observed. We further found theoretically that mass-action dynamics in local areas can be indicative of the outcome of virus spread in a spatially structured cell population. This suggests that previous insights, gained from the analysis of ordinary differential equations, remain relevant for the spread of viruses in a spatial setting.

## Methods

### Mathematical/computational approaches

Both an agent-based model and a metapopulation model were used to examine the spatial dynamics of virus spread through a population of growing target cells. Analytical and computational methods are described in detail in the Text S1.

### Experimental approaches

#### Cell culture and adenovirus infections

Human 293 embryonic kidney epithelial cells (HEK293 or Ad293 cells – Agilent Technologies) were grown in Dulbecco's modified Eagle's medium (DMEM) supplemented with 10% fetal bovine serum (FBS). All cells were cultured at 37°C, 95% humidity, and 5% CO2. HEK293-H2BmCherry cells stably express the core nuclear histone H2B fused to monomeric red fluorescent protein mCherry [40] and were selected for and maintained using 400 ug/mL G418 supplemented in the culture medium. A recombinant adenovirus type-5 (Ad5) that expresses enhanced jellyfish green fluorescent protein (EGFP), in which the EGFP gene driven by the immediate early promoter of human cytomegalovirus (CMV) was substituted into the E1A and E1B regions (AdEGFPuci), was previously described [39]. AdEGFPuci viral stocks, with titers of 10^9^–10^11^ pfu/ml, were obtained. The plaque assays were done as follows; 24 h prior to infections, 5×10^5^ Ad293 cells were seeded onto 60 mm gridded dishes. 24 h post seeding (cells density of ∼70–80% confluence) 1 ml of serially diluted AdEGFPuci stocks (∼10^2^–10^1^ pfu/ml dilutions) were applied directly to the cell monolayers and incubated while rocking at 37°C, 95% humidity, and 5% CO2 for 1–1.5 h. Subsequently, non-absorbed virus was aspirated, and the cell monolayer was overlaid with 3 ml of 0.65% agarose in 1× DMEM/10%FBS. AdEGFPuci infected cells were cultured under the above conditions (less rocking) for at least 20 days, and the cells were fed by overlay with 2 ml–0.65% agarose/1× DMEM/10%FBS every 3–4 days.

#### Quantification of AdEGFPuci plaque development

AdEGFPuci infected cells were scanned under UV illumination daily (100× magnification) using an Axiovert 200 M phase-contrast fluorescent microscope (Zeiss, Germany). Each grid was scored for the presence of green (virus-infected) cells and developing plaques were monitored for at least 20 days. Fluorescent images of AdEGFPuci infection that gave rise to plaques were recorded at 24–48 h intervals and montages of the recorded plaques were compiled using Microsoft PowerPoint. The rate of plaque expansion from AdEGFPuci infections was quantified by measuring the area of GFP fluorescence observed per day using Adobe Photoshop version 7.0. Briefly, each AdEGFPuci plaque image recorded was analyzed in Photoshop by selecting a green color range of moderate to low intensity to ensure the majority of the GFP expressing cells in the plaque was encompassed. The number of pixels displaying the selected color range of green fluorescence was defined as the area of GFP fluorescence in each plaque assayed. We analyzed several of the plaques at least three times to determine the average area of GFP fluorescence in each plaque/day. The area of GFP fluorescence from a single infected AdEGFPuci cell was quantified similarly and used to estimate the number of green (virus-infected) cells in the developing plaques. To calculate the number of fluorescent cells in the plaques using Photoshop, first the area of an individual cell fluorescing above a threshold was obtained from analyzing four independent cells at day 7 (the first day plaque was recorded). The mean area of fluorescence from the entire plaque was then divided by the mean area of fluorescence from an individual cell to derive the number of cells in the plaque. Standard deviations were then calculated by standard methods.

## Supporting Information

Text S1 Mathematical details underlying the results presented in this paper. This file contains details about mathematical and numerical methods that were used to analyze the computational models.
(PDF)
