# Supplementary material for: Complex Spatial Dynamics of Oncolytic Viruses In Vitro: Mathematical and Experimental Approaches
Source: PLoS Comput Biol. 2012 Jun 14;8(6):e1002547. doi: 10.1371/journal.pcbi.1002547 (PMC3375216; doi:10.1371/journal.pcbi.1002547)
Supplement: Text S1 — Mathematical details underlying the results presented in this paper. This file contains details about mathematical and numerical methods that were used to analyze the computational models. (PDF) [file pcbi.1002547.s001.pdf]

Complex spatial dynamics of oncolytic viruses in vitro: mathematical and experimental approaches

Dominik Wodarz, Andrew Hofacre, John W. Lau, Zhiying Sun, Hung Fan, and Natalia L. Komarova

Text S1

1 ODE description of the agent-based model

Alternative methodologies for studying agent-based model dynamics. In the paper, we considered an agent-based model where individuals can only interact with their nearest neighbors, i.e. interactions are spatially restricted. In the past, attempts have been made to describe spatial systems of this nature with ordinary differential equations (ODEs), using pair approximation methods [2, 11, 9, 10, 1]. We implemented those methods but found that this does not provide a sufficiently good description of the outcomes and the dynamics. The dynamics tended to fall somewhere between the outcome observed in the mass-action ODE (that is, the ODE describing the system where mass-action rules apply over the whole domain) and the true spatial dynamics observed in the simulations of the agent-based model. In fact, in many cases the pair approximation only provided a correction to a mass-action description, failing to describe the dynamics of a spatially-restricted system. More details about the accuracy of the pair approximation method will be provided in a subsequent paper [12]. Hence, this method was not used to study the spatial system and will not be further discussed. Instead, we obtained a full understanding of the spatial system through a combination of extensive numerical simulation, as described in the text, and analytical methods described here.

Another method which employs ODEs to describe spatially extended systems was proposed by [6, 7]. There, a stochastic metapopulation model was considered based on the Nicholson-Bailey and (unsaturated) Lotka-Volterra systems for natural enemies. The equations for the averaged values of the prey and predator contain a covariance term, and a moment closure procedure was used to approximate this term in the limit of very large migration rates (under the assumption of global migration dynamics). The effect of spatial structures was then studied for these systems. Our system is different from those of [6, 7] because it contains saturated prey growth, and thus possesses a natural stable equilibrium, thus making the role of spatial dynamics very different. The system in the present paper is also different in another respect. Refs. [6, 7] report that the results of the large migration rate approximation hold even in the case of moderate and small migration rates. For our system unfortunately this is not the case. Extensive simulations showed that the dynamics of metapopulations changes dramatically depending on the relative magnitude of migration rates. The results described in this paper do not fall under the umbrella of "large migration rate" limit. For this reason, we did not implement the analytic

methodology of [6, 7] to study spatial dynamics. Below we present an ODE description which we used for a stochastic mass-action system.

A mass-action ODE description. Let us turn off the spatial restrictions in the agent-based model, and allow each reproducing cell to attempt to place its offspring in a randomly chosen site anywhere in the grid. Reproduction is successful if the spot that was picked was empty. The same type of rule applies to infection events whereby an infected cell can in principle infect a target cell anywhere in the grid. The non-spatial system can be well described by ODEs, which are given as follows:

$$\frac{dS}{dt} = RS(1 - \frac{S+I}{K}) - \frac{BSI}{K}, \tag{1}$$
$$\frac{dI}{dt} = \frac{BSI}{K} - AI \tag{2}$$

where S denotes the number of susceptible hosts and I denotes the number of pathogen-infected hosts. Uninfected hosts divide with a rate R, and this growth is density-dependent, limited by the carrying capacity K. When an infected host is encountered by a susceptible one, infection occurs with a rate B. Infected hosts die with a rate A. This is a variant of the saturated Lotka-Volterra predator-prey model. In order to derive this system from the stochastic process, let us denote by $\phi_{i,j}(t)$ the probability that at time t, there are i infected and j uninfected hosts in the system. To derive the equations for expected numbers of infected and uninfected hosts, we consider the probability rates of various events. In infinitesimal time-interval, Δt , starting from state (i, j), the following changes can happen:

- An uninfected host can reproduce with probability $R(1 - (i + j)/K)\Delta t$, which contains the probability rate R for a given uninfected host to reproduce, times the probability that a randomly chosen spot will be empty, $1 - (i + j)/K$.
- An infected host can die with probability $A\Delta t$, where A is the probability of death.
- An infected host can pass infection to an uninfected host. For a given infected host this happens with probability $B\frac{i}{K}\Delta t$, where B is the infection rate and i/K is the probability to find an uninfected host by randomly placing infection in one of the grid-spots.

Under these assumptions, the equations for averages can be derived, which are given by

$$d\langle i\rangle/dt = R(\langle i\rangle - \langle i^2 + ij\rangle/K) - B\langle ij\rangle/K, \tag{3}$$
$$d\langle j\rangle/dt = B\langle ij\rangle/K - A\langle j\rangle, \tag{4}$$

where the angular brackets denote the expected values. The above equations involve the second moments, and as with most nonlinear stochastic processes,

the system is not closed. However, if we simply decouple the equations by replacing $\langle i^2 \rangle \to \langle i \rangle^2$ and $\langle ij \rangle \to \langle i \rangle \langle j \rangle$, and denote $S = \langle i \rangle$, $I = \langle j \rangle$, we obtain the non-spatial ODEs, equations (1-2) of the main text, which are the same as equations (1-2) above.

As an alternative approach, we also considered a higher-order moment closure method, which results in a system of 5 ODEs that contain higher correlations [8, 5]. We have checked numerically the mass-action system behavior against the predictions of the simple ODEs, as well as the larger, 5-equation system. We found remarkable correspondence with equations (1-2), which did not warrant the usage of the more complicated system.

System (1-2) is characterized by two equilibria. In the trivial case, the host population persists at carrying capacity, while the pathogen population is extinct, i.e. $S^{(0)} = K$, $I^{(0)} = 0$. Alternatively, the pathogen establishes a successful infection, and this equilibrium is described by $S^{(1)} = AK/B$, $I^{(1)} = RK(B-A)/B(R+B)$. The pathogen can establish a successful infection if its basic reproductive ratio, R_0, is greater than one, where $R_0 = B/A$. The approach to the coexistence equilibrium can be either monotonic, or can involve damped oscillations. Oscillations are observed if the basic reproductive ratio of the pathogen lies above the following threshold: $R_0 > \frac{1}{2}[1 + \frac{\sqrt{A(A+R)}}{A}]$. Note that pathogen-mediated extinction of the host population is not possible, as this system is deterministic. However, if the equilibrium number of susceptible host cells is less than one, or if oscillations drive the number of susceptible hosts below one, extinction is expected to occur in a corresponding stochastic system (see below). This system has been used to describe the outcomes of the non-spatial agent-based model in figure 1 of the main text.

2 One-dimensional stochastic metapopulation modeling

To ensure that our findings are not dependent on one particular modeling approach, we compare the results obtained from the agent-based model to those derived from a metapopulation model.

A metapopulation model is a different way to analyze dynamics in a spatial setting. It consists of a collection of n local patches. To set up the stochastic dynamics, we first write down a deterministic analogue of our model. It is given by the following system of ODEs:

$$\dot{S}_i = rS_i\left(1 - \frac{S_i + I_i}{k}\right) - \frac{\beta S_i I_i}{k} + \frac{m_S}{2}(S_{i-1} - 2S_i + S_{i+1}), \quad (5)$$

$$\dot{I}_i = \frac{\beta S_i I_i}{k} - aI_i + \frac{m_I}{2}(I_{i-1} - 2I_i + I_{i+1}), \quad 1 < i < n, \quad (6)$$

where r is the division rate of target cells, β is the infection rate, a is the death rate of infected cells, and k is the carrying capacity of one patch. We assumed

that the patches are arranged in a 1-dimensional linear array, and both target cells and infected cells can migrate to the neighboring patches to the left and to the right of a given patch with migration rates given by m_S and m_I respectively. To define the boundary conditions, we set

$$\begin{aligned}
\dot{S}_1 &= rS_1\left(1-\frac{S_1+I_1}{k}\right)-\frac{\beta S_1 I_1}{k}+\frac{m_S}{2}(-S_1+S_2), && (7)\\
\dot{I}_1 &= \frac{\beta S_1 I_1}{k}-aI_1+\frac{m_I}{2}(-I_1+I_2), && (8)\\
\dot{S}_n &= rS_n\left(1-\frac{S_n+I_n}{k}\right)-\frac{\beta S_n I_n}{k}+\frac{m_S}{2}(S_{n-1}-S_n), && (9)\\
\dot{I}_n &= \frac{\beta S_n I_n}{k}-aI_n+\frac{m_I}{2}(I_{n-1}-I_n), && (10)
\end{aligned}$$

which means that populations do not migrate away from the ends of the linear chain of patches.

To proceed with the definition of the stochastic metapopulation model, we adopt the so-called Gillespie algorithm [3, 4]. The simulation proceeds as a sequence of time-steps. At each time-step, let us denote the local numbers of susceptible and infected cells as $S_i^{(n)}, I_i^{(n)}$. The different terms in system of equation (5-6) define the relative probability weights of different events that could happen at the next update. Let us form the sum

$$\Sigma^{(n)}=\sum_{i=1}^{n}\left(rS_i^{(n)}\left(1-\frac{S_i^{(n)}+I_i^{(n)}}{k}\right)+\frac{\beta S_i^{(n)} I_i^{(n)}}{k}+aI_i^{(n)}+m_S S_i+m_I I_i\right).$$

Then the probability that at patch i, an infected cell will die is given by

$$aI_i^{(n)}/\Sigma^{(n)},$$

the probability that at patch i, an infection event will take place is given by

$$\beta S_i^{(n)} I_i^{(n)}/\Sigma^{(n)},$$

the probability that a target cell will migrate from patch i to patch $i+1$ is given by

$$(m_S/2)S_i^{(n)}/\Sigma^{(n)},$$

and so on. Guided by these probabilities, we pick the next event and update the system accordingly. For example, if the next event is a death of an infected cell in patch i, we take $I_i^{(n+1)} = I_i^{(n)} - 1$, and keep the rest of the variables the same. If the next event is an infection event in patch i, we take $I_i^{(n+1)} = I_i^{(n)}+1$, $S_i^{(n+1)} = S_i^{(n)} - 1$, and keep the rest of the variables the same. If the event is a migration of a target cell from patch i to patch $i+1$, we take $S_i^{(n+1)} = S_i^{(n)} - 1$, $S_{i+1}^{(n+1)} = S_{i+1}^{(n)} + 1$. Finally, we determine the length of the time-step between state $(n+1)$ and state (n) from the exponential distribution with the constant $\Sigma^{(n)}$: $P(\tau) = \Sigma^{(n)} e^{-\Sigma^{(n)}\tau}$.

A description of possible system outcomes in the metapopulation model is given in figure 9 of the main text. Here we note that although the topology of the phase space in the metapopulation and in the agent-based models is the same, and the outcomes that are observed are very similar, there are certain features of the metapopulation model that are different from the agent-based model. In particular, in the coexistence region (region MC), the dynamics of the metapopulation can proceed according to several different patterns. It is possible that local extinction occurs initially as the infected population starts spreading through space. During this initial spread, a small number of infected cells move into a space filled with susceptible target cells that exist close to carrying capacity; the ensuing dynamics result in target cell extinction. This occurs because of oscillatory dynamics which are promoted by a large initial number of target cells in the patch. When the infected cell wave catches up with the target cell wave and the two waves travel together, however, the situation becomes different: the patches into which the infected cells migrate are not filled with target cells close to carrying capacity anymore. Instead the target cells are present at significantly lower levels, and such initial conditions favor more stable dynamics with lower amplitude oscillations that are more likely to lead to persistence. This eventually gives rise to most patches containing persisting populations. Alternatively, if the virus is weaker, local populations already persist during the initial spread of the virus through space, leading to the absence of any traveling waves and the immediate persistence of most local populations.

A summary of all the outcomes of the metapopulation model (figure 9 of the main text) is provided below. They are based on the local equilibrium value of target cells, $S^{(1)}$, and infected cells, $I^{(1)}$, as well as the wave height, H, calculated by means of PDE analysis, see main text.

- If $I^{(1)} < 1$ (above and to the left of the black line), pathogen extinction, (outcomes MD and ME) is observed. Otherwise, the following outcomes can occur.

- Outcome MA (extinction of both populations without traveling waves) is observed if $S^{(1)} < 1$ (below the white line) and $H < 1$ (to the left of the green line).

- Outcomes MB or MB1 (traveling waves) are observed if $S^{(1)} < 1$ (below the white line, Figure 1c) and $H > 1$ (to the right of the green line).

- Outcome MC (coexistence) is observed if $S^{(1)} > 1$ (above the white line and below the black line).

Note, however, that the conditions $S^{(1)} < 1$ and $I^{(1)} < 1$ are only approximate. In the Gillespie simulations, population extinction tends to occur when the number of individuals approaches three instead of one, which is where the white line in Figure 9 of the main text was drawn, for the sake of clarity. Further note that these results depend on the assumption that the PDE provides a

good deterministic description of the metapopulation dynamics. Calculations [12] indicate that this is true if $m_S < 5r/12$.

3 Derivation of the initial growth-laws of infection

In this section we focus on the initial growth patterns exhibited by the agent-based model, as well as the in vitro system. It turns out that some approximate growth laws of infection can be derived based on first principles. Let us study the initial propagation of infection, and assume that the uninfected cells do not divide in the time-scale of interest.

The growth of a plaque. First, we assume that the virus spreads outward as a plaque, and does not leave uninfected cells behind. Let us denote by $\mathcal{A}$ the area of the circular plaque. The growth-law of the plaque in a two-dimensional culture is then

$$\dot{\mathcal{A}} = \gamma\sqrt{\mathcal{A}}, \tag{11}$$

because the rate of new infections is proportional to the circumference of the plaque, and that in turn is proportional to the square root of $\mathcal{A}$. The coefficient γ in this empirical model is proportional to the infectivity of the virus. The solution of this equation is given by

$$\mathcal{A} = \left(\frac{\gamma t}{2}\right)^2, \tag{12}$$

and the radius of the plaque is $r(t) = \gamma t/(2\pi)$.

Denote by z the distance from the center of the plague, and $\rho(z)$ the density of infected cells. The infected cells die at the rate a, and thus the density of cells decays exponentially in time, with the rate a. Let us suppose that the infection is introduced at time $t = 0$. A location at point with coordinate z gets infected at time $t_z = 2\pi z/\gamma$. If the current time is t, a location with coordinate z has been infected for the duration of time, $t - 2\pi z/\gamma$. Therefore, the density of infection at that point at time t is

$$\rho(z) = e^{-a(t-2\sqrt{\pi}z/\gamma)},$$

To obtain the total number of cells, we integrate over the plaque,

$$y(t) = \int_0^{2\pi}\int_0^{\gamma t/(2\sqrt{\pi})} \rho(z) z\, dz\, d\varphi = \frac{\gamma^2}{4\pi}\frac{e^{-at} - 1 + at}{a^2},$$

such that for small values of at, the plaque grows quadratically in time, $y(t) \sim t^2$, and for large values of at we observe a linear growth, $y(t) \sim t$.

This analysis can be generalized to 3D:

$$y(t) \propto \frac{2(1 - at - e^{-at}) + (at)^2}{a^3}.$$

Here, for small values of at we have a cubic growth, $y(t) \sim t^3$, and for large at we have a quadratic growth law, $y(t) \sim t^2$.

Disperse growth phenotype. Next, we consider a different mode of infection spread, where the wave of infection leaves uninfected cells behind. Let us denote by ρ_S and ρ_I the local densities of uninfected and infected cells, respectively. At dynamic equilibrium, ρ_S and ρ_I remain spatially uniform inside the infected region. They satisfy the local equations,

$$\dot{\rho}_S = L(\rho_S, \rho_I)\rho_S - \beta\rho_S\rho_I, \quad (13)$$

$$\dot{\rho}_I = \beta\rho_S\rho_I - a\rho_I, \quad (14)$$

where the reproduction rate $L(\rho_s, \rho_I)$ is proportional to the number of unoccupied spots in the neighborhood of a given cell, which in turn is proportional to $1-\rho_I-\rho_S$, yielding the logistic growth law for ρ_S. Inside the region of infection, these densities assume their equilibrium values, $\rho_S^{(1)}$, $\rho_I^{(1)}$. The total number of infected cells however will grow because at the edge of the infected region, the equilibrium is not reached, and new infections continue to happen. This happens proportionally to the number of infected cells at the edge. The area of the infected region satisfies equation (11), and the total number of infected cells is given by $A\rho_I^{(1)}$. This quantity grows as a square of t, see equation (12).

4 The fitting procedure

In the main text we present the result of a fitting procedure which shows that our model can closely reproduce the behavior of the *in vitro* system. Here we outline the details of the fitting procedure. The experimentally obtained time-series of the number of infected cells were compared with the genetic algorithm model. In order to find the model parameters, we ran the agent-based model on a 200x200 grid, starting from a specified initial condition (where the entire grid was initially filled with uninfected cells, and N_i infected cells were placed in the middle at day 7, with (a) $N_c = 81$ and (b) $N_c = 3$ for the ring structure and the disperse phenotype respectively. The dynamics with different parameters were simulated 50,000 times. For each individual simulation, the $\log_{10}$ of the parameters of the model were taken randomly from a uniform distribution between -4 and 0 (in other words, the parameters belong to the range $[10^{-4}, 1]$). The parameters thus varied were: the infection probability, B, the death probability of infected cells, A, and the linear division probability of the uninfected cells, R. The time-step of the simulation was fixed at 4.2×10^{-2} and 5.5×10^{-2} days for the two cases.

For each parameter combination, the simulation was performed 100 times, and the average growth-curve compared with the experimental time-series by means of the least squares procedure. The best fitting model for each of the two cases is presented in the paper.

References

[1] Boots, M. and Sasaki, A. 2002. Parasite-Driven Extinction in Spatially Explicit Host-Parasite Systems. The American Naturalist 159, 706–713.

[2] De Carvalho, K.C. and Tome, T. 2007. Stable oscillations of a predator-prey probabilistic cellular automaton: a mean-field approach. J. Phys. A: Math. Theor. 40, 12901–12915.

[3] Gillespie, D.T. 1976. A general method for numerically simulating the stochastic time evolution of coupled chemical reactions. J. Comp. Phys., 22, 403434.

[4] Gillespie, D.T. 1977. Exact stochastic simulation of coupled chemical reactions. J. Phys. Chem., 81, 23402361.

[5] Keeling, M.J. 2000. Multiplicative Moments and Measures of Persistence in Ecology. J. Theor. Biol. 205, 269–281.

[6] Keeling, M.J., Wilson, H.B. and Pacala, S.W. 2000. Reinterpreting Space, Time Lags, and Functional Responses in Ecological Models. Science 290, 1758–1761.

[7] Keeling, M.J., Wilson, H.B. and Pacala, S.W. 2002. Deterministic Limits to Stochastic Spatial Models of Natural Enemies. The American Naturalist 159, 57–80.

[8] Lloyd, A.L. 2004 Estimating variability in models for recurrent epidemics: assessing the use of moment closure techniques. Theoretical Population Biology 65, 49–65.

[9] Rand, D.A. Correlation Equations and Pair Approximations for Spatial Ecologies. 1999. CWI Quarterly 12, 329–368.

[10] Sato, K., Matsuda, H. and Sasaki, A. 1994. Pathogen invasion and host extinction in lattice structured populations. J. Math. Biol. 32, 251–268.

[11] Satulovsky, J.E. and Tome, T. 1994. Stochastic lattice gas model for a predator-prey system. Phys. Rev. E 49, 5073–5080.

[12] Sun, Z., Wodarz, D. and Komarova, N.L. 2011. On the accuracy of analytic approximations to agent-based natural enemy models. In preparation.
